# Hepatoprotective and Fat-Accumulation-Reductive Effects of Curcumin on Metabolic Dysfunction-Associated Steatotic Liver Disease (MASLD)

**DOI:** 10.3390/cimb47030159

**Published:** 2025-02-27

**Authors:** Jasmine Harumi Sabini, Kris Herawan Timotius

**Affiliations:** Faculty of Medicine and Health Sciences, Krida Wacana Christian University, Jakarta 11510, Indonesia; jasmine.sabini@ukrida.ac.id

**Keywords:** fatty liver, fibrogenesis, lipogenesis, lipophagy, lipolysis, NAFLD

## Abstract

Fat accumulation is the hallmark of metabolic dysfunction-associated steatotic liver disease (MASLD). Given the intimidating nature of its treatment, curcumin (CUR) emerges as a potential therapeutic agent due to its proven effectiveness in managing MASLD. This review aimed to evaluate previous reports on the hepatoprotective and fat-accumulation-reductive effects of CUR administration in preventing or treating MASLD. CUR administration can modulate serum liver enzymes and lipid profiles. The fat accumulation of MASLD is the primary cause of oxidative stress and inflammation. By reducing fat accumulation, CUR may attenuate the inflammation and oxidative stress in MASLD. In addition, CUR has been proven to restore the dysfunctional cellular energy metabolism capacity and attenuate fibrogenesis (antifibrotic agent). Their hepatoprotective effects are associated with fat accumulation in MASLD. Lipid metabolism (lipogenesis, lipolysis, and lipophagy) is correlated with their hepatoprotective effects. CUR has prophylactic and therapeutic effects, particularly in early-stage MASLD, primarily when it is used as a fat reducer. It can be considered an excellent natural therapeutic drug for MASLD because it protects the liver and attenuates fat accumulation, especially in the early stage of MASLD development.

## 1. Introduction

Fatty liver or metabolic dysfunction-associated steatotic liver disease (MASLD), previously well known as nonalcoholic fatty liver disease (NAFLD), is developing as a noteworthy worldwide public health problem. MASLD’s main characteristic is liver fat accumulation (steatosis). At a particular phase of MASLD, fat accumulates excessively in the liver. As a disease with multifactorial causes, MASLD is frequently associated with metabolic disorders like oxidative stress, inflammation, fibrosis, insulin resistance, and dyslipidemia [1]. Presently, polyphenol-rich dietary strategies for preventing and treating MASLD are promising. They usually have antioxidant and anti-inflammation properties that help manage metabolic diseases, including MASLD. Since oxidative stress and inflammation are the risk factors, consuming polyphenol-rich diets is recommended for treating MASLD [2,3]. One of the most familiar bioactive compounds in polyphenol-rich diets is curcumin (CUR), which is derived from turmeric. CUR is an acceptable nutraceutical and herbal ingredient for many diseases owing to its powerful antioxidant and anti-inflammation properties. It can inhibit and reduce fat accumulation. Therapeutically, CUR administration is a promising potential agent for MASLD therapy [4,5]. Recently, it was reported that fat accumulation is also associated with the bile acid and gut microbiota profiles. Curcumin can modulate gut bacteria’s dysbiosis and the intestinal barrier’s functional damage. Based on these capacities, curcumin can maintain a healthy gut–liver axis. Therefore, combined supplementation, curcumin, and probiotics are probably the best option for preventing fatty liver [6], even though there is still concern about their bioavailability. Improved systemic bioavailability of curcumin, particularly in combination with piperine, is a necessity for additional research [7].

Due to the presence of many emerging reports on the potential use of CUR to remedy MASLD, the authors composed this comprehensive review. After presenting the physiopathological phases of fat accumulation during MASLD progression (Section 2), we describe the serum biomarkers of MASLD (Section 3). Then, we describe and evaluate available information on the hepatoprotective roles of CUR lipogenesis and free fatty acid (FFA) influx (Section 4), lipolysis and lipophagy (Section 5), and finally, liver fibrosis (Section 6).

## 2. The Spectrum of MASLD: Stages, Pathology, and Therapeutic Insights

**Figure 1:**
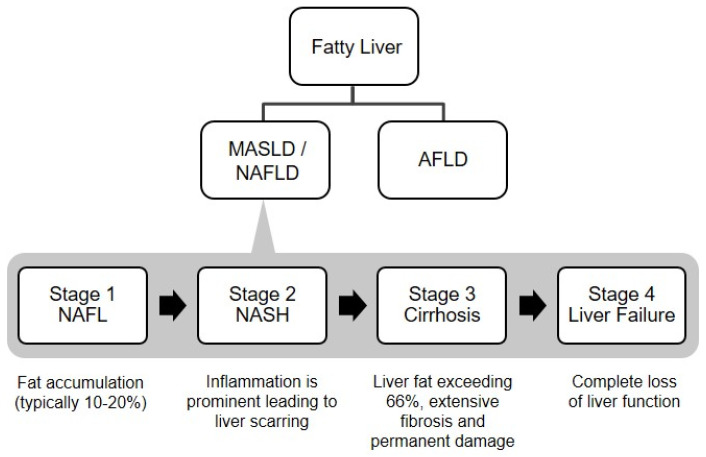
Classification and phases of MASLD.

A certain degree of fat storage is typical for the liver; a level exceeding 5 per cent of its weight categorizes it as fatty liver. A fatty liver might remain asymptomatic, but the accumulation of excess fat can initiate liver inflammation [8]. Fatty liver disease exhibits a fat deposit. It manifests in two primary forms: metabolic dysfunction-associated steatotic liver disease (MASLD) and alcoholic steatohepatitis or alcoholic fatty liver disease (AFLD) (Figure 1). MASLD pertains to excessive fat infiltration in the hepatocytes of individuals without a history of alcohol abuse. In contrast, AFLD is linked to significant alcohol consumption [9].

The primary feature of MASLD is the abnormal accumulation of fat or triglyceride in the liver. The term covers a spectrum of MASLD, starting with simple steatosis (NAFL—nonalcoholic fatty liver), characterized by hepatocellular steatoses alone without any liver damage observed. On the more severe end of the spectrum is nonalcoholic steatohepatitis (NASH), a condition involving necroinflammatory reactions and hepatocellular steatosis. NASH is a progressive condition that has the potential to develop into liver cirrhosis and hepatocellular carcinoma (HCC) [10].

MASLD progresses through four distinct phases, each posing varying levels of severity and potential complications (Figure 1). The initial stage, grade 1 or NAFL (nonalcoholic fatty liver), represents the early phase marked by fat accumulation. There is no inflammation or scarring, and symptoms may not be readily apparent, making detecting it challenging [10]. The external fat accumulation indicates grade 1 without impacting liver function significantly.

Progressing to grade 2, or NASH (nonalcoholic steatohepatitis), inflammation becomes a prominent feature. At this stage, fat makes up 10–20% of liver mass, possibly increasing to 34–66%. Chronic inflammation at grade 2 manifests as fibrosis, indicating scar tissue’s appearance. Progression to grade 2 is critical because liver fibrosis begins to replace healthy tissue, disrupting liver function and restricting blood flow, leading to further complications.

Grade 3, or cirrhosis, represents the most severe level of MASLD. The liver fat content is very high, exceeding 66%, accompanied by inflammation of the surrounding structures. Severe liver scarring characterizes this stage, where extensive scar tissue replaces healthy liver tissue, drastically disrupting function and blood flow. Cirrhosis poses an increased risk of liver failure (grade 4) and development of liver cancer.

The final stage, grade 4 or liver failure, indicates a complete loss of liver function. In liver failure, the organ can no longer effectively process toxins or drugs, leading to their accumulation in the body. This progressive sequence underlines the importance of identifying and treating MASLD early to lessen the risk of severe complications and recuperate long-term effects.

**Figure 2:**
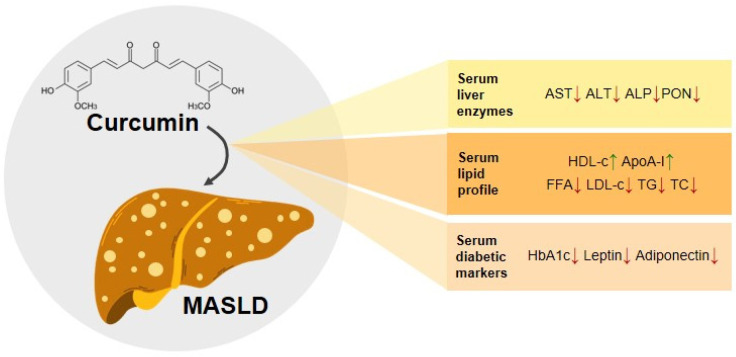
The possible role of curcumin in various MASLD-related fat metabolisms. The symbol ↑ means it is upregulated, while the symbol ↓ means it is downregulated.

Diferuloylmethane (1,7-bis(4-hydroxy-3-methoxyphenyl)-1,6-heptadiene-3,5-dione) is commonly referred to as curcumin (CUR). CUR, which serves as the key active component derived from turmeric, is widely utilized in Asian cuisine as a dietary spice and food coloring, and also has applications in beverage industries. CUR is accepted as an interesting nutraceutical and herbal ingredient for various diseases [4,5]. CUR has attracted attention because of its potential to prevent and reduce the early stages of MASLD. Research shows that administering CUR shows promise in preventing or alleviating hepatic steatosis, a hallmark of the early stages of MASLD. Additionally, CUR has demonstrated efficacy in preventing and ameliorating NASH, a progressive inflammatory condition, and reducing fibrosis [11,12]. In addition, research findings show that the administration of CUR can reduce the progression of NASH to hepatocellular carcinoma (HCC) [13]. The reported benefits of CUR include its ability to intervene at multiple stages of MASLD, making it a potential therapeutic agent with diverse effects on liver health. These insights underscore the potential of CUR as a natural intervention strategy in managing MASLD, promising prevention and attenuation in the early stages (Figure 2).

## 3. The Ability of Curcumin to Normalize the Serum Biomarkers of MASLD

The assessment of liver health includes monitoring serum liver enzymes, lipids, and diabetes biomarkers. Research findings showed that CUR can lessen the severity of MASLD, as expressed by normalizing serum liver enzymes, lipids, and diabetes markers (Figure 2) [14,15,16].

### 3.1. Curcumin Modulation of Serum Liver Enzymes

Two essential biomarkers of liver damage are aspartate aminotransferase (AST) and alanine aminotransferase (ALT). Their elevated concentrations indicate potential liver problems, especially in patients with MASLD. Alkaline phosphatase (ALP) further contributes to the assessment of cell leakage and loss of cell membrane integrity. ALT, AST, and ASP are serum biomarkers of hepatotoxicity, particularly MASLD. MASLD is a common cause of elevated hepatic transaminases [17]. The administration of CUR can normalize the serum biomarkers of liver injury, as indicated by the reduction in serum liver enzyme levels (ALP, ALT, AST) (Figure 2) [18,19,20,21].

Another essential serum biomarker in MASLD is paraoxonase (PON). PON is an antioxidant enzyme primarily found in the liver and has lipophilic–hydrolytic properties [22,23]. The PON family comprises three main types: PON1, PON2, and PON3. While PON1 and PON3 are predominantly expressed in the liver and secreted into the serum, PON2 is retained within the cell. In MASLD, both serum and liver PON activities are observed to decrease. Moreover, PON1 levels are significantly reduced in patients suffering from chronic liver diseases [24]. As an antioxidant biomarker, PON1 activity is a crucial indicator of MASLD. Increased serum PON activity signifies a decrease in liver oxidative stress [22,25].

PON shares a complex relationship with HDL. It inhibits the oxidation of HDL and LDL particles, alleviating the inflammation linked to oxidative stress. By preventing lipid oxidation, PON is crucial in reducing the risk of atherosclerosis and MASLD. A positive correlation was shown between PON activity and serum HDL-C concentration. The activity of the HDL-associated antioxidative enzyme, PON-1, is associated with low HDL cholesterol levels in MASLD [26]. CUR exhibits a hypolipidemic effect by enhancing PON activity in plasma [23]. Interestingly, the administration of CUR alone or together with metformin can increase the PON activity and, as a result, decrease the dyslipidemia [27].

### 3.2. Curcumin Regulation of Serum Lipid Profile

Along with serum liver enzymes (ALT, AST, and ALP), serum lipids, like FFA, TC, TG, LDL-c, and HDL-c are biomarkers of MASLD [17]. CUR has also been proven to improve the serum lipid profile by lowering the FFA, TC, TG, and LDL-c, and increasing HDL-c. This suggests that CUR positively affects liver lipid metabolism. Moreover, CUR influences lipid metabolism by increasing the plasma concentration of apolipoprotein A-I (ApoA-I), an essential component of HDL [14,23,28,29].

Moreover, serum bilirubin is a well-known marker for metabolic disorders, including MASLD and cardiovascular disease. There is a noted inverse relationship between MASLD and serum bilirubin levels, suggesting that serum bilirubin can serve as a protective marker against MASLD [30,31]. The serum levels of total bilirubin (TBIL), direct bilirubin (DBIL), and indirect bilirubin (IBIL) are inversely associated with MASLD. In particular, the IBIL level is inversely correlated with the onset and degree of NASH patients [32]. In this regard, CUR has been shown to promote the excretion of bilirubin in the bile [33].

### 3.3. Curcumin’s Effect on Serum Diabetes Markers

MASLD is often associated with diabetes. In this context, CUR has shown a significant impact on various markers associated with diabetes, such as levels of fasting blood sugar and haemoglobin A1c (HbA1c). It has the potential to improve glucose tolerance and insulin resistance and modulate the target genes of sterol regulatory element-binding proteins (SREBPs). Therefore, CUR can contribute to lowering fat accumulation and improving insulin sensitivity. CUR’s capacity to improve insulin resistance occurs through regulating the IRS/AKT pathway [34]. Monitoring glucose metabolism and insulin sensitivity by CUR is essential in managing MASLD.

Changes in serum adipokine levels have been observed in patients with MASFD. Adiponectin, a prominent adipokine, is correlated with reduced insulin resistance, fat accumulation, and inflammation. Plasma adiponectin released by adipocytes can reduce body fat and play an essential role in improving insulin sensitivity or reducing insulin resistance through increasing hepatic glucose production and fatty acid oxidation. Adiponectin receptors mediate these effects by activating AMPK and PPAR-α signalling pathways [23,35]. CUR treatment dramatically enhances serum adiponectin expression and improves insulin resistance [34,36].

**Figure 3:**
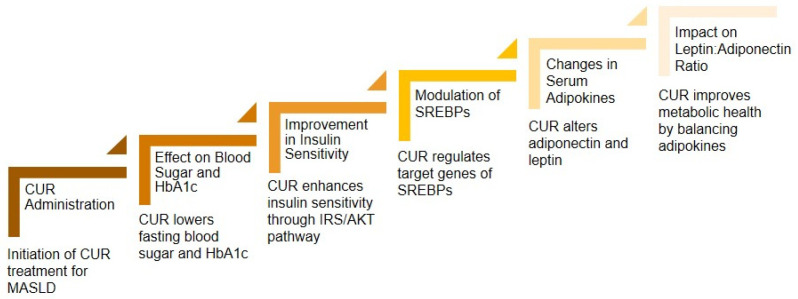
Curcumin’s impact on diabetes markers in managing MASLD.

CUR can reduce serum leptin levels in MASLD. Its administration can decrease the leptin–adiponectin ratio or regulate the balance of adipokines [37]. This ratio is an essential biomarker of dysfunctional adipose tissue; because of its lower leptin–adiponectin ratio, it is associated with better metabolic health and improved insulin sensitivity. This ratio might be a clinically helpful marker in diagnosing metabolic diseases, including MASLD (Figure 3) [38].

## 4. Hepatoprotective Role of Curcumin in De Novo Lipogenesis and FFA Uptake

Fat accumulation is closely related to an imbalance between the generation of fat (lipogenesis and FFA influx) and its removal or breakdown (lipolysis and lipophagy). Hepatic fat accumulation contributes to the progress of steatosis to NASH, NASH-related cirrhosis, and HCC [39]. Within MASLD, the increased hepatic fat content primarily exists in the form of neutral lipids stored within intracellular droplets known as lipid droplets [40]. Fibrosis occurs in the final phases of MASLD.

### 4.1. De Novo Lipogenesis (DNL) in MASLD

#### 4.1.1. Biosynthesis of Fatty Acids

The biosynthesis of fatty acids starts with acetyl-CoA and malonyl-CoA as starting substrates, forming triglycerides. The breakdown of glucose and fructose primarily causes acetyl-CoA. Various enzymes facilitate the conversion of these sugars into triglycerides. This excess acetyl-CoA can leave mitochondria and enter the cytoplasm as citrate. Moreover, ATP citrate lyase-degrading enzyme (ACLY), a key enzyme in the liver cells, can convert the citrate into acetyl-CoA and oxaloacetate. They become the primary substrate for new adipogenesis and contribute to fatty acid overproduction [41]. This conversion of citrate into acetyl-CoA links glucose breakdown to the biosynthesis of fatty acids [42].

Targeting the key enzymes involved in fatty acid biosynthesis, such as acetyl-CoA carboxylase (ACC), ACLY, citrate/isocitrate carrier (CIC), and fatty acid synthase (FAS), presents a promising therapeutic approach [43]. It has been shown that CUR can suppress these adipogenic enzymes, contributing to its hepatoprotective effects [41]. These inhibitory capacities are part of CUR’s hepatoprotective properties. In addition, CUR can also correct the dysregulated expression of ACLY, possibly through the AMPK-mTOR signalling pathway, and inhibit citrate transport and metabolism. As a result, CUR can help prevent or reduce fat accumulation by blocking citrate disposition [42].

#### 4.1.2. The Damage to Mitochondrial Biosynthesis Genes

The main pathophysiological characteristic of NASH is mitochondrial damage, particularly the downregulation of several mitochondrial biogenesis genes, including mitochondrial DNA (mtDNA). Several proteins encoded in the nucleus play crucial roles in the metabolic processes in mitochondria and the maintenance of mtDNA. MASLD patients can significantly deplete mtDNA, encoding numerous proteins essential in the electron transport chain (ETC) cycle. Damage to mtDNA can disturb the ETC chain and ATP production [44].

Several transcription factors play crucial roles in regulating de novo lipogenesis (DNL). The damaged mtDNA can decrease the expression of transcription proteins. The expression of transcription proteins includes mitochondrial transcription factor A (Tfam), nuclear respiratory factors 1 and 2 (Nrf1 and Nrf2), PPAR coactivator-1 (PGC1), and PPAR-α. Tfam, Nrf2, and PGC-1α decrease in MASLD [20,45]. Interestingly, the three main transcription factors that regulate DNL are sterol regulatory element-binding protein 1c (SREBP-1c), liver X receptors (LXRs), and carbohydrate response element-binding protein (ChREBP). They are upstream stimulatory factors (USFs) [46,47]. CUR can suppress the expression of these transcription factors that significantly attenuate DNL and reduce fat accumulation [42]. Excessive production of LXR stimulates DNL, leading to hypertriglyceridemia by activating SREBP-1c. CUR can also reduce the expression of LXR-α and SREBP-1c, indicating its specificity in targeting key regulators of fat biosynthesis [20,41].

However, CUR can reverse the dysfunction of mitochondria bioenergetics and normalize the expression of several mitochondrial genes. As a result, CUR can increase ATP concentration and elevate the transcriptional factors responsible for regulating mitochondrial biogenesis, not only PGC1α, but also Nrf1 and Tfam. They are responsible for the lower mitochondrial respiratory chain activity and ATP production [48].

CUR can also decrease fat accumulation by downregulating sbp-1, fat-6, and nhr-49 mRNA levels. SBP-1, a homolog of SREBPs, plays a crucial role in DNL as it regulates stearoyl-CoA desaturase upstream. The suppression of sbp-1 and fat-6 can lead to a decrease in fat accumulation. Stearoyl-CoA desaturase is responsible for the critical step in converting saturated fatty acids to monounsaturated fatty acids. The nhr-49 gene, which encodes a functional homolog of PPARs, also regulates stearoyl-CoA desaturase upstream [49].

#### 4.1.3. The Role of Signalling Pathways in Lipogenesis

Several signalling pathways are associated with DNL, such as SIRT1, Akt/GSK-3β, and AMPK/SIRT. SIRT1 is influenced by nicotinamide adenine dinucleotide (NAD(+)). As a key metabolite, NAD(+) is involved in energy metabolism for non-redox NAD(+)-dependent enzymes and redox reactions. It regulates dehydrogenase activity in various catabolic processes. However, prolonged NAD(+) homeostasis imbalance is related to MASLD. In this context, CUR is believed to regulate NAD(+) metabolism, thereby enabling the prevention and treatment of MASLD. CUR can increase NAD(+) and SIRT3, improving mitochondrial function. Therefore, CUR has a potential role in the therapeutic strategy for diseases associated with NAD(+) imbalance [50].

CUR’s effects on MASLD also extend to regulating the Akt/GSK-3β signalling pathway. CUR involves activating Akt and inhibiting GSK-3β, a critical measure to prevent liver damage by mitigating oxidative stress and inflammation [51]. Moreover, GSK-3β inhibition by Akt can prevent fibrosis by inhibiting the production of extracellular matrix components involved in fibrotic tissue formation. A multifaceted approach involves ameliorating liver damage by suppressing oxidative stress and inflammation through modulating the Akt/GSK-3β pathway [52].

The activation of AMPK has emerged as a protective mechanism for MASLD and has shown the ability to reverse insulin resistance. AMPK’s activation induces beneficial effects, including increasing fatty acid oxidation and inhibiting hepatic lipogenesis [53,54].

Considering its potential therapeutic implications for MASLD, the activation of AMPK promoted by CUR has received significant attention. CUR has been shown to increase hepatic total AMPK protein levels while suppressing FAS and phosphatide phosphohydrolase-related activities, inhibiting DNL [54,55]. Studies showed that the attenuation of steatosis could be achieved by activating AMPK [56,57].

**Figure 4:**
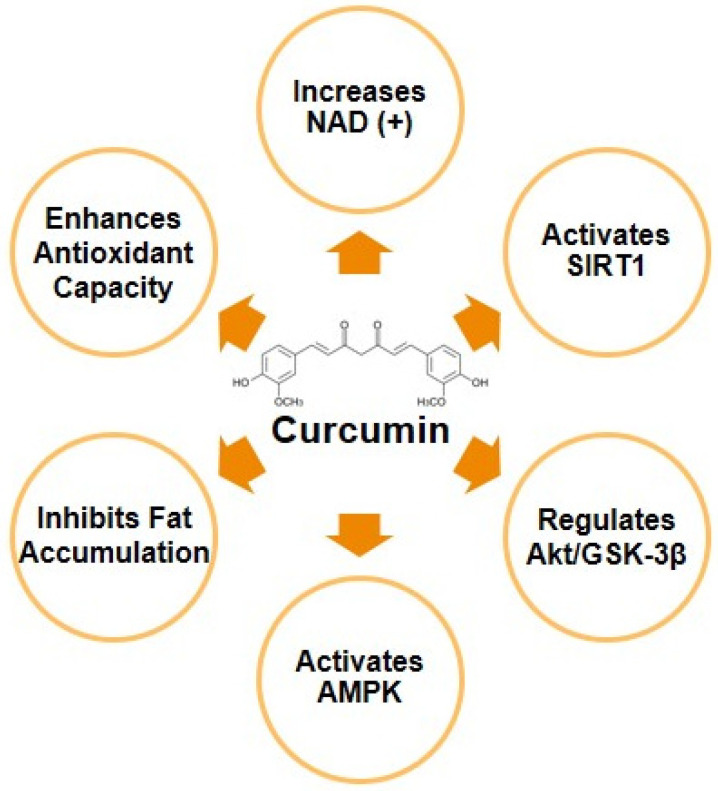
Curcumin’s role in regulating signalling pathways for MASLD.

AMPK plays a critical role in oleic acid-induced DNL. As an effective modulator, CUR can suppress oleic acid-induced fat accumulation. CUR can also inhibit 11β-HSD1, which activates the AMPK/SIRT1 signalling pathway, restoring fat homeostasis. Furthermore, CUR has been demonstrated to increase phosphorylated AMPK while reducing FAS, SREBP-1, ACC, and HMGCR, thus improving fatty acid catabolism and antioxidant capacity (Figure 4) [55,58,59].

#### 4.1.4. The Role of Particular Hormones in Lipogenesis

Certain hormones can promote and inhibit DNL. Under insulin and leptin resistance, high dietary fructose consumption significantly contributes to developing hepatic steatosis into MASLD. CUR can improve insulin and leptin sensitivity and effectively ameliorate insulin and leptin signalling defects by reducing the expression and activity of protein tyrosine phosphatase 1B (PTP1B) [60]. CUR can phosphorylate both the insulin receptor and the insulin receptor substrate 1 (IRS-1), which enhances the insulin signalling pathway and elevates the levels of p-Akt and extracellular signal-regulated kinase 1/2 (ERK1/2) [61,62]. High blood glucose and insulin levels can trigger signalling pathways that activate transcription factors. These pathways, including the DNA-dependent protein kinase (DNA-PK) and AKT-mTOR pathways, regulate the post-translational modifications of transcription factors [46].

### 4.2. Free Fatty Acid Influx into the Liver

The influx of FFA is another critical process in DNL. Elevated levels of FFAs, which can result from increased lipolysis or an imbalance in FFA flux, are critical pathogenic factors in hepatic steatosis [57]. FFA influx into the liver is possible through several mechanisms, including passive diffusion, facilitated transport, and receptor-mediated endocytosis. The amount of hepatic fatty acids derived from DNL contributes about 26% of the total hepatic fatty acids in MASLD, in contrast to about 5% in healthy individuals [63].

An increased amount of FFAs causes the overproduction of TG-rich lipoproteins such as serum VLDL. Excess FFAs can enter mitochondria and then β-oxidation. The β-oxidation process begins with converting cytoplasmic FFAs into fatty acyl-CoA that is transported into the mitochondria facilitated by carnitine palmitoyltransferase 1 (CPT-1). Once fatty acyl-CoA is within the mitochondrial matrix, it undergoes degradation, becoming acetyl-CoA by β-oxidation [64].

The excess FFAs in the mitochondria can also impair the electron transport chain (ETC). Electrons can interact with oxygen to form ROS as the ETC is impaired. Moreover, the increasing mitochondrial membrane permeability dissipates the membrane potential and reduces ATP synthesis [65]. A significantly impaired ETC in NASH needs to be normalized. The regular ETC activity progressively decreases during the progression of MASLD. On the other hand, the β-oxidation in mitochondria is increased in NAFL and NASH. The imbalance of mitochondrial β-oxidation and ETC will result in the overproduction of ROS due to the electron leakage from ETC [66].

CUR treatment significantly increases β-oxidation [23,57,67]. Combined with vitamin E, CUR can upregulate mRNA expression of CPT1a, Nrf-1, PPAR-α, and Tfb2m, which suggests it enhances the oxidation of fatty acid and biogenesis of mitochondria [68].

## 5. Hepatoprotective Role of Curcumin on Lipid Droplet Metabolism

Most liver fat is present as lipid droplets (LDs) in hepatocytes. These droplets are encapsulated by a single layer of phospholipids surrounding a core occupied by neutral lipids, mainly triacylglyceride (TAG) and sterol esters. The polar phospholipid heads face the cytosol, while the nonpolar acyl chains are occupied in the hydrophobic lipid core. Various proteins, including perilipin, are on the LD capsule surface. There are two primary mechanisms for LD degradation, namely lipolysis and lipophagy.

### 5.1. Lipolysis

Lipolysis, the process of enzymatically breaking down lipids in the cytosol, is primarily facilitated by neutral lipase, the primary catalyst for TG degradation. The process begins with the hydrolysis of TG by adipose triglyceride lipase (ATGL), resulting in diacylglycerol (DG). This DG is then hydrolyzed by hormone-sensitive lipase (HSL), which has been phosphorylated by perilipin to produce monoacylglycerol (MG). In the final step, MG is hydrolyzed by monoacylglycerol lipase (MGL), yielding glycerol and FFA [69,70].

Lipolysis is under the influence of several hormones, including catecholamines (which stimulate it), natriuretic peptides (which also stimulate it), and insulin (which inhibits it). These hormone receptors can express proteins that regulate the final steps in hormone signalling to lipolyses, such as ATGL, HSL, and preplan. An increase in lipolysis can lead to elevated levels of FFAs. The regulation of lipolysis by hormones and the expression of proteins that regulate lipolysis are critical factors in reducing fat accumulation [69,70].

**Figure 5:**
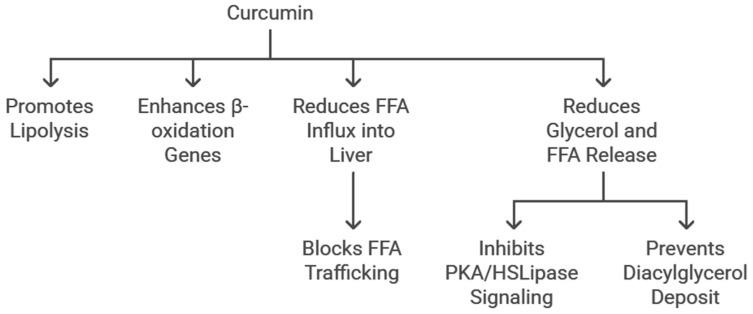
The role of curcumin in balancing lipolysis stimulation and inhibition.

CUR can promote lipolysis and the expression of β-oxidation genes. It can reduce the influx of FFAs into the liver by blocking FFA trafficking. It can reduce glycerol and FFA release by blocking protein kinase (PK)A/hormone-sensitive lipase lipolysis signalling and by blocking FFA trafficking and preventing the depositing of diacylglycerol (Figure 5) [71,72].

### 5.2. Lipophagy

Lipophagy is an autophagic/lysosomal pathway and is a fat metabolism via the lysosomal degradative autophagy pathway [73]. The most essential lipase in lysosomes is lysosomal acid lipase (LAL). In lipophagy, autophagosomes fuse with lysosomes, leading to the breakdown of LD, which then releases FFA and glycerol. These byproducts are then absorbed by autophagosomes and transported to lysosomes, where they undergo further degradation through acidic hydrolases. Lipophagy is crucial in maintaining lipid homeostasis and is a prospective therapeutic target for MASLD [74]. It participates in both the anabolism and catabolism of fats. By producing FFA and eliminating surplus lipids, lipophagy helps to preserve lipid equilibrium [75].

In the liver, lipophagy operates through various molecular pathways. These include the SIRT1-lipophagy pathway, the interaction of perilipins (PLINs) with lipophagy, and the role of ATGL in lipophagy. Additionally, the PPAR-α-lipophagy pathway is involved, as well as the nuclear receptor, farnesoid X receptor (FXR), which functions in contrast to PPAR. Specifically, PPAR-α promotes lipophagy, whereas FXR inhibits the initiation of autophagy. Thus, PPAR-α and FXR exert opposing regulatory influences [75].

The liver, abundant in lysosomes, is a primary site for autophagy. When autophagy is amplified, it leads to an increase in the consumption of liver fat and a reduction in the accumulation of liver fat. Both autophagy and fat metabolism have a reciprocal influence on each other and play a vital role in preserving the balance of fat metabolism. Furthermore, feedback from fat metabolism can trigger or halt autophagy in response to various irregular fat metabolisms, liver damage repair, and other pathological alterations, thereby controlling fat metabolism [75].

**Figure 6:**
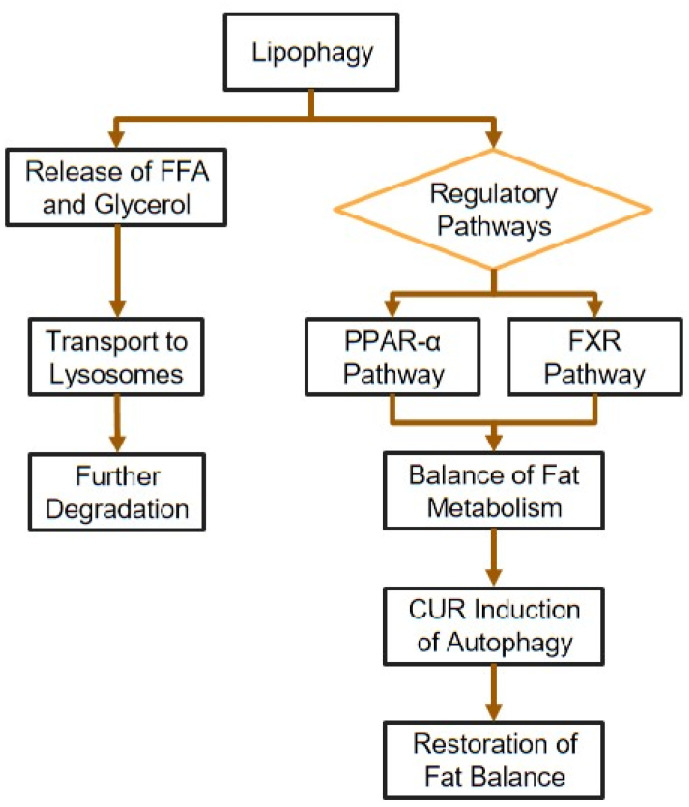
The role of curcumin in lipophagy.

In this context, CUR can induce autophagy and stimulate fatty acid-induced autophagy via mTOR-dependent pathways in hepatic stellate cells (HSCs) [76,77]. CUR can potentially help restore the balance of fats in the liver of MASLD patients by triggering both autophagy and lipophagy (Figure 6). Another necessary process is macroautophagy, where the cell forms an autophagosome that engulfs and breaks down fat droplets. Interestingly, while CUR does not affect the overall autophagy process, it can significantly increase the levels of a specific protein involved. Furthermore, an extended study has shown that CUR5-8, a synthetic CUR derivative, can increase the LC3-Atg7, a marker of autophagy formation [78].

Although CUR has been widely studied for its potential metabolic benefits, most of the existing research focuses on in vitro and in vivo experimental models exploring its molecular mechanism. Determining an optimal dose remains a challenge due to variations in its bioavailability, metabolism, and study methodologies. To bridge these gaps, further research is needed to establish clear dosing guidelines and long-term efficacy in the context of MASLD.

## 6. Hepatoprotective Effects of Curcumin as an Antifibrotic Agent

In MASLD, DNL is one of the primary sources of hepatic fats, leading to fat accumulation that creates cellular pressure, damage, and fibrosis. The appearance of scar tissue indicates liver fibrosis due to chronic inflammation at grade 2. Liver fibrosis begins to replace healthy tissue, disrupting liver function and restricting blood flow, leading to further complications. The liver comprises various cell types working together for its diverse functions. Understanding the molecular mechanisms of fibrogenesis in MASLD is crucial for developing urgently needed antifibrotic therapies [79,80,81]. Hepatic cells, including hepatocytes responsible for metabolic functions, form close relationships, producing signalling molecules that regulate their activity through intercellular crosstalk [82].

In MASLD, crosstalk between immune and non-immune cells, such as Kupffer cells (KCs), hepatic sinusoidal endothelial cells (LSECs), and HSCs, plays a significant role in disease stages, integrating multiple systems driving inflammation and fibrosis [83,84]. KCs, situated in hepatic sinusoids, act as tissue macrophages, contributing to liver regeneration and immune surveillance. LSECs form the endothelial lining of hepatic sinusoids, influencing the vascular structure and essential functions for nutrient exchange. Additionally, in the Disse space, apart from the sinusoid, HSCs regulate blood flow and maintain the extracellular matrix [85,86]. Each cell type plays a crucial role in response to repeated liver damage, leading to inflammation, scar tissue formation, and tissue structure loss, ultimately culminating in liver failure [79].

### 6.1. Curcumin Effects on Kupffer Cells and Monocyte-Derived Macrophages

Hepatic macrophages have central roles in the pathogenesis of chronic liver damage. They are potential targets for combating fibrosis. This diverse immune cell population displays remarkable heterogeneity and performs various critical functions for maintaining liver homeostasis and responding to injury. In the liver, its cellular heterogeneity is partly due to the diverse macrophage origins [87].

Hepatic macrophages originate from two origins that create the liver’s immune environment. First, intrahepatic macrophages originate from circulating monocytes that are attracted and recruited to the liver injury in response to chemokine signalling. Second, they may be derived from locally self-renewing embryonic-derived macrophages, KCs. KCs are able to sense tissue injury and initiate inflammatory responses. Meanwhile, macrophage infiltration derived from Ly-6C(+) monocytes is associated with chronic inflammation and fibrogenesis, suggesting involvement in the pathogenesis of liver pathology [87].

#### 6.1.1. Resident Macrophage: Kupffer Cells

In a steady state, as liver macrophages, KCs maintain a clean environment by removing pathogens and debris cells, ensuring immune tolerance, and releasing anti-inflammatory cytokines [88]. In liver fibrosis, KCs emerge as important regulators and actively play an essential role in developing and regressing MASLD pathological conditions. KCs play a dynamic role during liver injury by rapidly releasing soluble mediators (e.g., oxidants, cytokines, and proteases) into the liver environment. These mediators profoundly affect HSCs, influencing essential processes such as proliferation, migration, and differentiation, promoting liver fibrosis [89,90].

The activation of KCs, which is stimulated by toxic lipids, is a critical step in NASH amelioration due to their ability to recruit other immune cells. However, the damage inflicted on KCs by toxic lipids may also increase lipid peroxidation. Interestingly, it has been indicated that the depletion of KCs can offer therapeutic benefits by ameliorating fibrosis [17].

KCs exhibit different polarization forms, mainly M1, representing a proinflammatory phenotype, and M2, associated with an immunoregulatory profile. KCs’ differentiation into the M1 phenotype is primarily induced by pathogen-associated molecular patterns (PAMPs) after interacting with Toll-like receptors (TLRs). The M1 phenotype can trigger the secretion of several proinflammatory cytokines, such as CCL2, CCL5, IL-1β, IL-12, and TNF-α. These cytokines cause liver injury that releases damage-associated molecular patterns (DAMPs). DAMPs will then interact with TLRs and thus amplify KC activation and inflammation. Furthermore, the CCL2 and CCL5 cytokines trigger the activation of HSCs, which initiates the fibrogenic response. The toxic lipids activate KCs that upregulate TLRs and enhance the lipopolysaccharide (LPS) response. On the other hand, KCs with M2 phenotype can produce anti-inflammatory cytokines like IL-4, IL-10, IL-13, and TGF-α [91].

Notably, CUR treatment inhibits M1 polarization of KCs and suppresses hepatic mRNA IL-1β and TNF-α [92]. CUR can also upregulate PPAR-γ and suppress the proinflammatory phenotype (M1) of KCs while promoting its anti-inflammatory phenotype (M2) polarization. In this context, CUR can reduce the liver damage and cell apoptosis induced by inhibiting the activation of the NF-κb pathway that depends on the distinct upregulation of PPAR-γ in KCs [93]. Therefore, PPAR-γ upregulation by CUR reduces oxidative stress and inflammation.

Activated KCs exhibit various functions that influence the development of MASLD. In particular, these cells secrete transforming growth factor β1 (TGF-β1), a critical factor in the progression of NASH to liver fibrosis. TGF-β1 promotes HSC activation, which leads to the excessive production of fibrogenic material. Continuous exposure to TGF-β1 from KCs facilitates the ongoing activation of HSCs [94,95]. The TGF-β1 affecting HSCs will be explained in more detail below.

#### 6.1.2. Infiltrating Macrophage

The proliferated local or recruited macrophages accumulate in the injured liver. During fibrosis regression, monocyte-derived cells undergo differentiation into Ly6C low-expressing ‘restorative’ macrophages. These specialized macrophages are crucial in resolving injury and developing targeted therapies for liver injury, including fibrosis. This regulation can occur through various means, such as the recruitment of specific monocyte subsets, the increased polarization of repaired macrophages, or the modulation of various macrophage effector functions [87].

Prophylactic CUR treatment significantly protects against liver inflammation and fibrosis, which aligns with these insights. This protective effect has been mainly observed in reducing the intrahepatic infiltration of monocytes. This reduction in monocytes is achieved by inhibiting the activation of KCs, subsequently suppressing the chemokine secretion crucial for initiating inflammation and fibrosis. Due to the suppression of chemokine secretion, CUR also prevents the polarization of macrophages toward M1. This dual impact on infiltrating macrophages highlights the potential of CUR as an innovative and effective therapeutic intervention for liver damage and fibrosis [96].

### 6.2. Liver Sinusoidal Endothelial Cells (LSECs) in Liver Fibrosis and Regeneration

The vital role of LSECs lies in sustaining the capillary network that provides crucial nutrient and oxygen delivery to the liver. Nutrients and oxygen are intricately linked to various processes, including scavenger function, autophagy, cellular senescence, mechanotransduction, and maintaining hepatic homeostasis by regulating inflammation and immune response [85,97,98]. Endothelial cells play a vital function in the zonation of the liver structure. They keep HSCs and hepatocytes quiescent in maturity, showcasing their significance in liver rejuvenation and chronic disorders by coordinating paracrine cell activities [99].

During liver injury, the capillarization of LSECs involves the transformation of a typical sinusoidal architecture into a more rigid and fibrotic structure. This structure is related to the development of fibrosis, typified by the damage of endothelial features of sinusoids. This remodelling of the hepatic vasculature is part of a larger dynamic that includes fibrosis and angiogenesis. In this case, fibrosis and angiogenesis are interconnected in a bidirectional manner. On the one hand, fibrosis often leads to areas of hypoxia in the liver, stimulating angiogenesis as a compensatory mechanism to improve oxygen delivery. On the other hand, the newly formed vessels may participate in the extension of fibrosis [100].

The complex process of angiogenesis has emerged as a significant contributor and has shown the ability to produce critical angiogenic factors. Hypoxia-inducible factor-1α (HIF-1α) and vascular endothelial growth factor (VEGF) are particularly interesting among these angiogenic factors. HIF-1α is an oxygen-sensitive transcription factor and essential in cellular responses to hypoxia. In the context of angiogenesis, LSECs may increase the HIF-1α expression under a hypoxic environment and initiate the transcriptional activation of genes, including the gene encoding VEGF. VEGF is a crucial regulator of angiogenesis that can promote endothelial cell proliferation, migration, and angiogenesis [99].

Surprisingly, CUR treatment can protect against fibrosis-associated angiogenesis and sinusoid capillarization via HIF-1α inhibition [101,102]. Combined with resveratrol, CUR can downregulate mRNA expression of HIF-1α and VEGF, thereby attenuating MAFLD, at least partly, by modulating HIF-1 signalling pathways [103]. Recently, curcumol, a derivative of CUR, was found to regulate LSEC angiogenesis. This process effectively suppresses angiogenic markers by targeting Krüppel-like factor 5 (KLF5) transcription factor. In a mouse liver fibrosis model, the CUR derivate curcumol (30 mg/kg) can efficiently inhibit angiogenesis by modulating LSEC autophagy and suppressing KLF5 expression [104].

Furthermore, LSECs are involved in more than just angiogenesis. LSECs induce hepatocytes proliferation during liver regeneration, while in fibrogenesis, they undergo morphological and functional changes, influencing HSCs activation [98,99]. Existing evidence indicates that LSECs lose their specialized phenotype before HSC activation and fibrosis development. Liver X receptors (LXRs) produced by LSECs influence the HSCs’ activation and contribute to liver fibrosis pathogenesis [105]. In the case of skin fibrosis, LXRs can hinder fibrosis by suppressing the infiltration of macrophages and reducing the release of the profibrotic cytokine IL-6. The expression of these receptor subtypes reveals that LXRα is mainly expressed in tissues with high-fat metabolism and is associated with fibrosis in MASLD [106].

LXRα plays a crucial role in MASLD via its regulation of fat metabolism, interacts with specific signalling pathways that promote fibrosis (profibrotic cytokines and growth factors) and engages in crosstalk with nuclear receptors affecting fibrotic gene expression. CUR treatment can reduce LXRα [107,108]. A high-fructose diet can increase the expression of LXR, SREBP-1c, ACC, and FAS. Upon CUR treatment, the hepatic expression of LXRα decreased [41]. The reduction of LXRα may also affect the underlying pathogenesis of fibrosis through HSCs’ activation. Although there are still minimal studies on the effect of CUR on LSECs’ and HSCs’ contribution in MASLD, CUR may inhibit HSCs’ fibrosis initiation through the downregulation of LXR-α gene transcription.

### 6.3. Hepatic Stellate Cells

HSCs are essential for liver development, regeneration, and tissue homeostasis. However, when activated by injury, HSCs transform into a profibrotic state. Several factors can mediate HSC activation. They are ROS, monocyte chemoattractant protein 1 (MCP-1), hepatocyte growth factor (HGF), CXC chemokine receptor type 4 (CXCR4) and CC-Chemokine Ligand 2 (CCL2). CUR treatment can lower TNF-α, IL-6, and MCP-1 [20]. When activated, HSCs enter a state that leads to the overproduction of the extracellular matrix by upregulating alpha-smooth muscle actin (α-SMA), collagen, proteoglycans, and glycoproteins such as laminin and fibronectin 2. This shift also involves the downregulation of matrix metalloproteinases (MMPs), contributing to angiogenesis in liver fibrosis [109,110]. The secretion of cytokines and chemokines by activated HSCs directly stimulates hepatocyte proliferation and acts indirectly through the interaction with LSECs or KCs to further promote the regenerative process. Therefore, HSCs represent a key central contributor to the development of liver fibrosis. On the other hand, inactivated HSCs have an essential role in the regression of liver fibrosis, emphasizing the double-edged nature or the dual role of HSCs in the liver [111].

During liver injury, HSCs play a dual role in liver fibrosis. On the one hand, they release HGF, promoting hepatocytes to proliferate. On the other hand, these same HSCs can inhibit hepatocyte proliferation and even induce apoptosis by releasing TGF-β. TGF-β can activate SMAD-related proteins, particularly SMAD3 in HSCs, promoting fibrogenesis [112]. TGF-β can also be produced by KCs or even hepatocytes themselves, contributing to the complex interaction in the progression of MASLD, making it a signalling target for managing liver fibrosis [113]. CUR treatment can improve liver fibrosis by diminishing TGF-β1 signalling [83,114,115]. In liver fibrosis induced by CCL4, CUR treatment can reduce the levels of α-SMA mRNA and collagen deposition in the liver. This effect can be achieved by suppressing TGF-β1 expression and inhibiting SMAD2/3 activation. CUR also increases SMAD7 expression, which acts as an inhibitory factor in the SMAD signalling pathway [112,116].

In combating MASLD and targeting HSCs, CUR is a promising intervention in easing liver fibrosis by targeting succinate signalling. When taken orally, CUR works to counteract the breakdown of fatty acids in the mitochondria, thereby reducing succinate formation in the liver. This reduction is achieved by blocking the activity of succinate dehydrogenase (SDH), demonstrating its ability to inhibit liver fibrosis. In HSCs, CUR prevents the induction of HIF-1α triggered by succinate. The accumulation of succinate in the liver is signal-promoting liver fibrosis via HIF-1α induction, a process that CUR counteracts. To sum up, CUR achieves a dual effect by reducing succinate buildup and preventing HSC activation through the succinate/HIF-1α signalling pathway [117].

CUR employs various pathways to prevent HSC activation during hepatic fibrogenesis. Activated HSCs play a crucial role in fibrosis by producing collagen [118]. CUR enhances the activation of PPARγ and inhibition of HSCs [119,120,121,122]. These, in turn, attenuate sinusoidal angiogenesis in liver fibrosis. LDL may also cause the activation of HSCs. However, CUR can counteract this process by activating PPAR-γ, which not only suppresses the gene expression of SREBP-2 and LDL receptors but also induces the expression of SREBP-1c. The multifunctional role of CUR in inhibiting HSCs activation and its consequential impacts on hepatic fibrogenesis show its potential as a potent therapeutic agent.

## 7. Conclusions and Future Perspectives

CUR administration is a promising additional or complementary therapeutic approach to preventing and treating MASLD by reducing fat accumulation. It can ameliorate lipogenesis and FFA influx and enhance lipolysis and lipophagy in the liver. In addition, it can also ameliorate hepatic fibrosis as a sign of the latest phase of MASLD. Its hepatoprotective role depends on its multi-target action on the pathological mechanism of MASLD. CUR exhibits significant potential in managing MASLD through multiple signalling pathways, including SIRT1, Akt/GSK-3β, and AMPK/SIRT. By regulating NAD(+) metabolism, CUR enhances mitochondrial function and mitigates oxidative stress and inflammation, thereby reducing liver damage and fibrosis. Additionally, CUR suppresses key adipogenic enzymes and promotes β-oxidation, preventing excessive fat accumulation in the liver. Its role in autophagy and the inhibition of hepatic stellate cell activation further supports its hepatoprotective properties. Collectively, these mechanisms highlight CUR as a promising therapeutic strategy for MASLD treatment and prevention.

Although promising, CUR therapeutic limitations include poor bioavailability due to low solubility, rapid metabolism in the gut and other organs, and limited systemic absorption. Therefore, future research may lead to improvements in CUR bioavailability, such as by conducting trials for new dosage forms and adjuvants to improve the delivery systems, to then determine an optimal dosing guidelines and long-term efficacy in MASLD.

Additionally, further clinical trials are essential in assessing the safety, effectiveness, and long-term impact of these innovative formulations in MASLD patients. Future investigations should also consider alternative administration methods, such as intravenous or transdermal delivery, which could help circumvent first-pass metabolism and improve systemic absorption. Overcoming these challenges may enhance CUR’s therapeutic potential, making it a more viable treatment option for MASLD and other metabolic conditions.

## Abbreviations

AMPK, adenosine monophosphate-activated protein kinase; CCL, chemokine ligands; CUR, curcumin; DNL, de novo lipogenesis; FAS, fatty acid synthase; FFAs, free fatty acids; GSK-3β, glycogen synthase kinase 3 bet; HSCs, hepatic stellate cells; IL, interleukin; LSEC, liver sinusoidal endothelial cell; LXR, liver X receptor; MASLD, metabolic dysfunction-associated steatotic liver disease; KCs, Kupffer cells; NASH, nonalcoholic steatohepatitis; Nrf, nuclear factor-erythroid 2-related factor; PON, paraoxonase; PPAR, peroxiproliferator-activated receptor; SREBP, sterol regulatory element-binding protein; TG, triglyceride; TGF, transforming growth factor.
